# A Comprehensive Review of the Potential Use of Green Tea Polyphenols in the Management of COVID-19

**DOI:** 10.1155/2021/7170736

**Published:** 2021-12-03

**Authors:** Trina Ekawati Tallei, Fatimawali, Nurdjannah Jane Niode, Rinaldi Idroes, B. M. Redwan Matin Zidan, Saikat Mitra, Ismail Celik, Firzan Nainu, Duygu Ağagündüz, Talha Bin Emran, Raffaele Capasso

**Affiliations:** ^1^Department of Biology, Faculty of Mathematics and Natural Sciences, Sam Ratulangi University, Manado, North Sulawesi 95115, Indonesia; ^2^Pharmacy Study Program, Faculty of Mathematics and Natural Sciences, Sam Ratulangi University, Manado, North Sulawesi 95115, Indonesia; ^3^Department of Dermatology and Venereology, Faculty of Medicine, Sam Ratulangi University, Manado, North Sulawesi 95115, Indonesia; ^4^Department of Pharmacy, Faculty of Mathematics and Natural Sciences, Universitas Syiah Kuala, Kopelma Darussalam, Banda Aceh 23111, Indonesia; ^5^Department of Pharmacy, Faculty of Pharmacy, University of Dhaka, Dhaka 1000, Bangladesh; ^6^Department of Pharmaceutical Chemistry, Faculty of Pharmacy, Erciyes University, Kayseri 38039, Turkey; ^7^Faculty of Pharmacy, Hasanuddin University, Makassar, South Sulawesi 90245, Indonesia; ^8^Department of Nutrition and Dietetics, Faculty of Health Sciences, Gazi University, Emek, Ankara 06490, Turkey; ^9^Department of Pharmacy, BGC Trust University Bangladesh, Chittagong 4381, Bangladesh; ^10^Department of Agricultural Sciences, University of Naples Federico II, 80055 Portici, Naples, Italy

## Abstract

Green tea is produced from *Camellia sinensis* (L.) buds and leaves that have not gone through the oxidation and withering processes used to produce black and oolong teas. It was originated in China, but its cultivation and production have expanded to other Eastern Asian countries. Several polyphenolic compounds, including flavandiols, flavonols, flavonoids, and phenolic acids, are found in green tea and may constitute greater than 30% of the dry weight. Flavonols, especially catechins, represent the majority of green tea polyphenols. Green tea polyphenolic compounds have been reported to confer several health benefits. This review describes the potential use of green tea polyphenols in the management of coronavirus disease 2019 (COVID-19). The immunomodulatory, antibacterial, antioxidant, and anti-inflammatory effects of green tea polyphenols have also been considered in this review. In addition to describing the bioactivities associated with green tea polyphenols, this review discusses the potential delivery of these biomolecules using a nanoparticle drug delivery system. Moreover, the bioavailability and toxicity of green tea polyphenols are also evaluated.

## 1. Introduction

Plants are a powerful medicinal resource, and numerous studies on the therapeutic value of plants have been conducted [1–4]. Some plants have the potential to be developed into anti-severe acute respiratory syndrome coronavirus 2 (SARS-CoV-2) therapeutic agents [5, 6]. One plant that has been used since ancient times for medicinal purposes is tea (*Camellia sinensis* (L.)). After further study, scientific evidence suggests that tea has many health benefits, which have been attributed to the presence of polyphenols contained in tea leaves. However, the polyphenols contained in tea leaves depend on the preparation of the leaves. White, yellow, green, black, oolong, and dark teas are among the different types of tea that are currently prepared for consumption [7]. Additionally, the benefits of tea are also contributed by other constituents contained in the leaves, including tannins, flavonols, and flavonol glycosides, alkaloids such as theobromine and caffeine, polysaccharides such as D-rhamnose, L-arabinose, D-galactose, and D-glucose, and minerals such as aluminum (Al), barium (Ba), calcium (Ca), copper (Cu), iron (Fe), potassium (K), magnesium (Mg), manganese (Mn), sodium (Na), strontium (Sr), titanium (Ti), and zinc (Zn) [8, 9].

Historically, people in the Western world more commonly prefer black tea, whereas people in Asia commonly prefer green tea [10]. However, green tea has recently gained global popularity due to reports that green tea contains more naturally preserved polyphenols [11]. Green tea provides a wide range of health benefits, including antiviral, antibacterial, and anti-inflammatory activities. Green teas have also been reported to combat or decrease the risk of cancer and improve brain function, in addition to neuroprotective, antianxiety, cardiovascular disease preventive, cholesterol-reducing, antiarthritic, and antiangiogenic impacts.

Green tea contains polyphenols, such as flavandiols, flavanols, and flavonoids, which are phenolic acids and represent greater than 30% of the total dry weight. Flavonols represent the majority of polyphenols found in green tea. Green tea is typically available as a liquid or in the form of powdered green tea extracts that contain various quantities of polyphenols (45%–90%) and caffeine (0.4%–10%). The major flavonoids found in green tea are primarily catechins, which are found in greater amounts in green teas than in black or oolong teas [12]. Green tea contains 4 major catechins: (–)-epicatechin (EC), (–)-epicatechin-3-gallate (ECG), (–)-epigallocatechin (EGC), and (–)-epigallocatechin-3-gallate (EGCG) [13].

Green tea polyphenols have antiviral properties, mediated through a variety of mechanisms, which is essential during a pandemic situation, as researchers are racing to find treatments against coronavirus disease 2019 (COVID-19). EGCG attaches to the viral hemagglutinin, preventing the virus from binding to the cell's target receptor, which prevents the virus from spreading. EGCG also changes the virus's envelope, preventing the virus from infecting other cells. L-theanine is also found in green tea, which stimulates human gamma delta T lymphocytes to produce interferon-*γ* (IFN-*γ*), an antimicrobial cytokine [14, 15]. The promising benefits of green tea polyphenols for the treatment of COVID-19 are highlighted in this review article.

## 2. Green Tea Polyphenols

Polyphenols, xanthine, theanine, inorganic salts, and other trace elements are among more than 200 compounds contained in green tea leaves [16]. Green tea polyphenols constitute 30% of the total dry leaf mass and represent the primary constituents of green tea leaves [17, 18]. Catechins are the most abundant polyphenols found in green tea leaves and are believed to be responsible for the wide range of bioactivities that have been observed in studies of green tea [19]. The polyphenol concentrations in the green tea products used in clinical trials have varied from 200 to 1207 mg, according to a meta-analysis [20]. Among the catechins found in green tea, EGCG is the most plentiful, representing approximately 60% of the total catechin concentration, followed by EGC (approximately 20%), ECG (approximately 14%), and EC (approximately 6%) [21].

EGCG has received the greatest attention and has been found to present antiviral-like biological [22], antimicrobial [23], and anticancer [24] activities. EGCG also has the potential to inhibit cellular lipid metabolism. Powdered green tea (matcha) has 137 times the amount of EGCG found in loose-leaf green tea (sencha) [25].

## 3. Green Tea: Matcha (Powdered Green Tea) versus Sencha (Loose-Leaf Green Tea)

A strong culture of tea drinking exists in Japanese society, which is manifested in the form of a tea ceremony [26]. Matcha and sencha are two varieties of green tea commonly used among the Japanese population. Both forms of green tea are derived from the same plant species, *Camellia sinensis*; however, their textures and flavors are vastly different [27].

Sencha is made from tea plants that have been exposed to the sun all year, whereas matcha is made from tencha, which come from plants that have been cultivated in the shade for 30 days prior to harvesting [28]. Tea farmers in Japan discovered this method of harvesting tea leaves for matcha accidentally after covering the tea leaves to prevent them from freezing during the winter. For most of the plant's growth, high-grade matcha is cultivated in the shade [29]. The shading of tea plants used to grow matcha can be performed using a variety of materials, including straw or bamboo mats. Shading increases the chlorophyll concentrations in the leaves, causing the tea leaves to turn dark green. This method of covering the leaves also boosts the amino acid contents, giving matcha its distinct umami flavor [30]. To avoid oxidation, the tea leaves used to make both matcha and sencha must be steamed [28].

Stems consisting of a shoot and two or three opened leaves are typically selected by hand for use in sencha tea. The sencha leaves are then steamed after being dried in humid air to maintain their freshness. Sencha is then pressed, dried, and prepared for sale. Unlike sencha, the tea leaves for matcha are selected from among the tencha tea plant's youngest leaves. The selected leaves are the two leaves at the end of the shoot's tip. The leaves are steamed immediately after they are harvested to maintain their color and nutrient contents. Tencha leaves are then ground into the very fine matcha powder using a specialized granite grinding wheel [31].

The consumption of matcha involves the consumption of the complete tea leaf, which provides more health benefits from the components of the tea leaves. Matcha is more costly than sencha because it is more difficult to produce. Furthermore, high-quality matcha can only be grown in specific areas. The quality of tea is largely determined by the soil and other environmental conditions in which the plant grows, resulting in a variety of unique metabolites [32]. Matcha powder has a bright, vibrant, green color, with the distinctive smell of raw tea leaves, and high-grade matcha has a sweet smell. The appearance should be very smooth and not lumpy.

## 4. Medicinal Use of Green Tea Polyphenol

### 4.1. Antiviral Activities of Green Tea Polyphenol against SARS-CoV-2

Recent studies have reported that the antiviral medication remdesivir was able to complex with the crystal structure of the SARS-CoV-2 RNA-dependent RNA polymerase (RdRp) protein, which may provide a basis for the pharmacological blockade of critical viral proteins, effectively preventing SARS-CoV-2 infection. They demonstrated the recognition of template primer RNA by the polymerase enzyme [33]. In the past, scientists have reported that green tea catechins were able to inhibit neuraminidase, disrupting the membrane of the influenza virus [34]. Another study [35] indicated that viral replication could be inhibited by ECGC through the control of the cellular oxidation-reduction environment [36], suggesting that the natural flavonoids may be able to antagonize the proliferation of SARS-CoV-2 [37–40].

The apparent lack of cytotoxic effects, even when polyphenols are used at considerably high concentrations, renders them potential antiviral drug candidates. Polyphenols are less toxic than other drugs, which makes them active antiviral candidates. A large number of polyphenols have been characterized, including theaflavin (TF1), theaflavin-30-O-gallate (TF2a), theaflavin-30-gallate (TF2b), theaflavin 3,30-digallate (TF3), myricetin, EGCG, hesperidin, and quercetin. Polyphenols have been assessed for their potential to serve as SARS-CoV-2 RdRp inhibitors. The active site of SARS-CoV-2 RdRp was bound by selected polyphenols in docking experiments, indicating their potential to serve as inhibitors [41]. EGCG was reported to obstruct porcine respiratory and reproductive infection with syndrome virus (PRRSV) when administered both before and after infection, and a total concentration of 125 *μ*M EGCG was sufficient to entirely prevent viral cell infectivity [42].

Another study examined the molecular docking of 18 phytoconstituents with seven different coronavirus proteins. The results of phytoconstituent docking were compared against the docking outcomes of the antiviral drugs remdesivir and chloroquine. The study found that EGCG binds to viral proteins more strongly than the reference drugs, chloroquine and remdesivir, and thus has greater antiviral efficacy [43]. Another study discovered that treating SARS-CoV-2 with green tea, roasted green tea, or oolong tea significantly reduced the viral infectivity. More remarkedly, EGCG significantly inactivated SARS-CoV-2 [44].

Antiviral drug research often primarily focuses on the inhibition of SARS-CoV-2 M^pro^, which is an important component involved in viral replication. As a result, many studies have been performed with the aim of identifying a powerful M^pro^ inhibitor. Eight polyphenols were isolated from green tea leaves that were found to present potent antiviral properties [45].

### 4.2. Antimicrobial Activities of Green Tea Polyphenol

Bacterial copathogens have been identified in a number of different types of viral infections and have significant impacts on both disease severity and mortality [46]. This coinfection has also been observed in patients with COVID-19 [47, 48]. Damage to ciliated cells has been associated with respiratory syncytial virus infection, which may lead to decreased mucociliary clearance, allowing for the increased bacterial adhesion to mucins, resulting in the increased bacterial colonization of the airway [49]. Patients with extreme SARS-CoV-2-associated pneumonia were associated with a 28% incidence of bacterial coinfection during intensive care unit (ICU) admission, with the most commonly identified bacteria including *Streptococcus pneumoniae*, *Haemophilus influenzae*, *Staphylococcus aureus*, and *Enterobacteriaceae* [50]. Therefore, critically ill patients who test positive for COVID-19 must be carefully monitored for the presence of bacterial coinfections.

Green tea polyphenols have demonstrated activity against a wide spectrum of microbes. Green tea polyphenolic catechins, especially EGCG and ECG, have been shown to impede the growth of a broad range of Gram-negative and Gram-positive bacterial species with moderate potency [21, 51, 52]. Damage to the bacterial cell surface is thought to be one mechanism through which galloylated catechins mediate antibacterial effects [53, 54]. The physical properties of the phosphatidylcholine (PC) and phosphatidylethanolamine (PE) bilayers were significantly altered by the compounds [53]. ECG disrupts the D-alanyl esterification of teichoic acid found in the *S*. *aureus* cell wall [54]. After *Bacillus subtilis* was incubated in a medium containing EGCG, EGCG was found to be deposited on the outer surface of the cytoplasmic membrane, suggesting that EGCG might inhibit the activity of many membrane proteins found in the cell envelope, including the oligopeptide ATP-binding cassette (ABC) transporter (Oppa), the phosphotransferase system (PTS) transporter, penicillin-binding protein 5 (PBP5), and the phosphate ABC transporter [55].

The ability of EGCG to inhibit the cytoplasmic enzyme dihydrofolate reductase is thought to be responsible for its antibacterial activity against *Stenotrophomonas maltophilia*, a Gram-negative opportunistic pathogen of increasing importance [56]. ECG, by contrast, has been shown to increase the release of lipoteichoic acid from the cell wall. Lipoteichoic acid, which is anchored in the staphylococcal cytoplasmic membrane, is known to modulate methicillin-resistant *Staphylococcus aureus* (MRSA) sensitivity against *β*-lactam antibiotics [57]. Bacterial cell membrane damage impairs the ability of bacterial cells to bind to host cells and similarly inhibits the ability of bacteria to bind other bacterial cells, preventing the formation of biofilms. The ability of bacterial cells to bind other cells is extremely important for the development of bacterial pathogenesis. Furthermore, bacterial membrane damage can disrupt the ability of bacterial cells to secrete toxins.

### 4.3. Anti-Inflammatory Activities of Green Tea Polyphenols

SARS-CoV-2-induced inflammatory and immune dysregulation and the pathophysiology of rheumatoid arthritis have been predicted to be similar based on the outcomes of a gene ontology study [58]. SARS-CoV-2 infection causes the dysregulation of 18 primary cytokines, which are highly expressed in symptomatic patients [59]. Previous research identified these inflammatory cytokines as TNF-induced apoptosis-inducing ligand (TRAIL), macrophage colony-stimulating factor (M-CSF), growth-regulated oncogene-*α* (GRO-*α*), granulocyte (G)-CSF, IL-2, IL-6, IL-7, IL-8, IL-10, IL-18, *β*-nerve growth factor (*β*-NGF), monocyte chemoattractant protein-1 (MCP-1), Skp1-cullin 1-F-box (SCF), IFN-*γ*-induced protein 10 (IP-10), platelet-derived growth factor-BB (PDGF-BB), IFN-*γ*, and IL-2 receptor *α* (IL-2R*α*) [58]. Although nonsteroidal anti-inflammatory drugs are a popular treatment for inflammatory disorders, they are associated with a number of side effects, including gastric inflammation, which can contribute to the development of gastrointestinal mucosal injury and peptic ulceration [60, 61]. As an alternative, researchers have searched for natural sources that might contain compounds with strong anti-inflammatory properties, high effectiveness, and a low risk of severe side effects.

It has been reported previously that EGCG had the potential to inhibit inflammatory signals, suggesting that it may be used as a broad-spectrum therapeutic in COVID-19 patients who are asymptomatic or symptomatic [58]. Additionally, green tea and its main ingredient EGCG have been reported to display anti-inflammatory properties in cellular, animal, and human studies [62] through the suppression of nuclear factor-kappa B (NF-*κ*B) activation [63], leading to the decreased expression of inflammatory cytokines and inflammation-mediated enzymes, such as TNF-*α* [64], COX-2 [65], and MMP-9 [66]. The expression of Toll-like receptor 4 (TLR4) and TLR2 is suppressed by EGCG through the inhibition of mitogen-activated protein kinase (MAPK) and NF-*κ*B signaling, resulting in the inhibition of proinflammatory cytokine production [67, 68]. The expressions of TNF-*α*, IL-1, and IL-8 were suppressed by treatment with a green tea petiole extract [69]. EGCG can suppress IL-8 production in the epithelium of human respiratory tract, reducing the severity of the inflammatory response [70]. Additionally, EGCG alleviates skin inflammation and asthma in rats stimulated with airborne fine dust particles [71]. In rat cell culture, EGCG has been shown to significantly reduce histamine release by 90% [72].

Green tea catechins appear to be responsible for cell migration, inhibition, and analgesia, and the inhibition of inducible nitric oxide synthase (iNOS) and COX-2 is known to decrease vasodilation and vascular permeability, respectively [73]. The administration of green tea extract prevents the release of systemic proinflammatory cytokines, neutrophil infiltration, and the expression of IL-6 and intercellular adhesion molecule-1 (ICAM-1) after hemorrhage/resuscitation-induced liver injury [74]. In a study examining the effects of green tea polyphenol ingestion on IL-2^−/−^ mice, 100 ml of green tea, containing 200–500 mg of polyphenols, reduced the incidence of colitis by reducing spontaneous INF-*γ* production [75]. Thus, green tea and its catechin constituents have strong anti-inflammatory properties, which may have potential to reduce the risk of developing severe COVID-19 symptoms.

### 4.4. Antioxidant Effects of Green Tea

Over the last few decades, an accumulation of scientific evidence has indicated that free radical damage plays a pathogenic role in respiratory virus infections. Surprisingly, very few medical professionals discuss the critical role of free radical damage in COVID-19 [76]. A strong link exists between proinflammatory factors and ROS in various lung diseases, including coronavirus infection, which is associated with both inflammation and oxidative stress. During critical illnesses, oxidative stress increases, contributing to organ failure. An intense inflammatory response, known as a cytokine storm, occurs in COVID-19, which may be mediated by oxidative stress [77]. After the virus replicates in the airways, the innate immune response activates dendritic cells and macrophages via TLRs and nucleotide-binding oligomerization domain (NOD) receptors to prevent the generation of inflammatory cytokines and ROS. The subsequent spread to the bloodstream has two consequences: (1) ROS and other inflammatory factors damage erythrocytes, resulting in the production of free iron and heme; and (2) activated neutrophils and macrophages generate respiratory bursts, producing H_2_O_2_ and superoxide radicals which cause oxidative stress [78].

Green tea catechins are natural antioxidants that tend to inhibit cellular injury and provide other benefits. These substances can reduce the formation of free radicals in the body, protecting molecules and cells from damage. Free radicals contribute to aging and a variety of diseases. Green tea polyphenols have been shown to act as direct antioxidants in vitro by scavenging ROS and chelating transition metals. An in vivo study suggested that catechins may act indirectly by upregulating phase II antioxidant enzymes [79], as well as enhancing the detoxification activity of enzymes, such as catalase, glutathione reductase, and glutathione peroxidase [80]. According to a previous report, catechins have a higher antioxidant capacity than glutathione, vitamin C, and flavonoids, indicating their critical role in cellular redox homeostasis [81].

Antioxidants can mitigate or prevent cell damage caused by free radical reactions. Antioxidants neutralize radical molecules through their scavenging abilities, which can halt chain reactions [82]. Antioxidants, combined with agents that inhibit the deleterious effects of lipid mediators and cytokines, may be useful for treating viral diseases. These agents could not only relieve disease symptoms but also mitigate the long-term effects of chronic oxidative stress, which has been associated with viral infections [83]. COVID-19 severity has been linked to increased oxidative stress and inflammation, particularly in the presence of chronic diseases associated with antioxidant system fragility [84]. Therefore, treatment with antioxidants may represent an effective method for reducing and preventing the excessive inflammation associated with the high oxidative state observed in COVID-19 [77].

### 4.5. Immunomodulatory Effects of Green Tea

COVID-19 infection is likely to be associated with immune dysregulation, particularly in critically ill patients, necessitating the development of novel treatment approaches. The impairment of innate immunity results in the disruption of various signaling pathways, resulting in increased proinflammatory cytokines, decreased interferons, depleted natural killer cells, and activated ROS production. The disruptions of these pathways have detrimental effects on the body's ability to combat infectious diseases and can contribute significantly to disease progression [85]. Thus, immunomodulatory treatments may be necessary for the management of COVID-19 to regulate the immune system by enhancing innate responses or suppressing inflammatory reactions.

EGCG and EGC, which are widely found in green tea, have the ability to act as immunomodulators by influencing T lymphocyte proliferation and cytokinin production. According to research, green tea extract can increase lymphoblast proliferation, inducing lymphocyte production, and EGCG is able to stimulate the production of IL-1*α*, IL-1*β*, monocytes, and lymphocytes [86]. Green tea extract has also been shown to act as an immunomodulatory agent in immunocompromised patients infected with *Candida albicans* by enhancing IL-8, IL-17A, and human *β*-defensin expression [87].

According to a study, green tea has an immunostimulatory effect in the cyclophosphamide-induced murine model of immunosuppression, possibly through the stimulation of cellular and humoral immune function, which increases total leukocyte counts [88]. These findings support the hypothesis that green tea may serve as a promising candidate immunomodulator that is capable of preserving immune function homeostasis under disease conditions. Using another murine model, green tea extract was shown to decrease antigen-specific IgE production by increasing the proportion of CD4+ CD25+ regulatory T (TR) lymphocytes in the spleen, suggesting that green tea extract may play a role in the regulation of the allergic response [89].

Green tea extract and EGCG have been shown to improve arthritis symptoms, including pathological arthritic features and serum CII-specific IgG2a antibody levels in animal models of arthritis. Furthermore, EGCG treatment significantly reduced inflammation-mediated cytokine production, including IL-6, IFN-*γ*, and TNF-*α*, while increasing IL-10 production [90].

Calgarotto et al. [91] reported a substantial decrease in ROS in BM CD34+ cells after a 30-day treatment, bolstering the assumption that green tea can protect cells and tissues from oxidative damage by scavenging oxygen-free radicals. Green tea also appeared to reduce the quantity of TR immunosuppressive cells and the level of CXCR4 expression in TR cells, potentially reversing the suppressor profile of the bone marrow microenvironment. According to one study [86], green tea can help alleviate the symptoms and pathology of autoimmune diseases in animal models. Additionally, EGCG was found to suppress autoreactive T cell proliferation, decrease the production of proinflammatory cytokines, decrease T helper type (Th) 1 and Th17 cells, and increase the TR population in lymphoid tissue and the central nervous system. EGCG has been suggested as a potential therapeutic agent for immune diseases, although human research data remains to be collected. However, EGCG and EGC have distinct effects on immune cells, and additional research remains necessary to determine the optimal ratio of these two compounds in green tea extract to determine what combination will result in the best immunomodulatory effects.

### 4.6. Effects of Green Tea Polyphenols on Reduced Mucin Hypersecretion

Mucus is an essential component of respiratory physiology, which acts as a physical barrier against inhaled particles and microbes. Excessive inflammation during conditions such as COVID-19 can lead to excessive mucus production, which can block the airway. Mucus accumulation can also result in chronic airway infection, further obstructing the airway [92]. Expectorants are recommended as prophylactic and therapeutic agents during COVID-19 therapy because they can increase the water contents of respiratory mucus, reducing the likelihood of airway blockage [93]. A mucus diluent, guaifenesin, has been reported to effectively clear mucus from the airways of COVID-19 patients [94]. Statins have also been shown to be effective in patients with COVID-19 [95].

In their research, Liang et al. [96] found that EGCG was able to ameliorate airway mucus production in rats, likely through the inhibition of epidermal growth factor receptor (EGFR) signaling pathways, suggesting that EGCG may serve as a therapeutic agent to prevent or treat chronic airway inflammation and abnormal airway mucus production. The antioxidative effects of EGCG on mucus secretion can be partially attributed to its ability to reduce hydrogen peroxide and superoxide levels, resulting in reduced mucus development.

**Figure 1:**
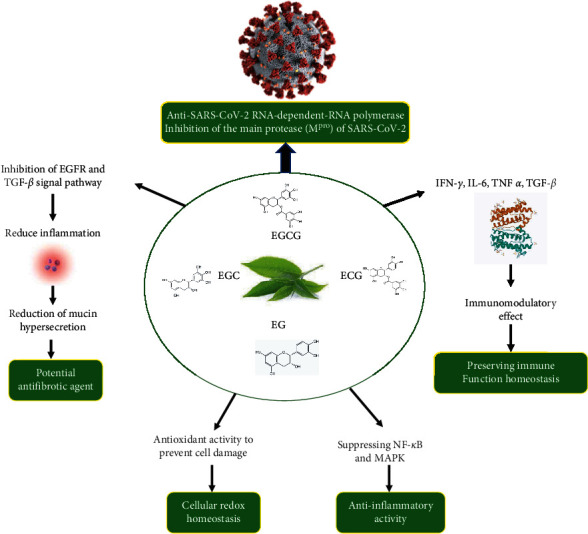
Potential use of green tea polyphenols in the management of COVID-19. EC: (–)-epicatechin (EC), ECG: (–)-epicatechin-3-gallate, EGC: (–)-epigallocatechin, EGCG: (–)-epigallocatechin-3-gallate, EGFR: epidermal growth factor receptor, IFN: interferons, IL: interleukins, TNF: tumor necrosis factor, TGF: transforming growth factor, MAPK: mitogen-activated protein kinase, NF-*κ*B: nuclear factor kappa-light-chain-enhancer of activated B cells.

The number of neutrophils in the respiratory tract increases during viral infection [97]. Transforming growth factor-*β* (TGF-*β*) is thought to recruit and activate neutrophils, in addition to prolonging neutrophil survival [98]. Inflammation and sensitization can occur due to neutrophil migration and activation in the pulmonary organs, which can lead to fibrosis and edema. Proinflammatory cytokines, including TNF-*α*, IL-6, and IL-1, induce an uncontrolled rise in active TGF-*β*, leading to the rapid and massive development of edema and fibrosis, which remodels and eventually blocks the airways, leading to lung functional failure [99, 100]. ECGC can reduce the activation of the TGF-*β* signaling pathway that triggers inflammation [101] and is expected to reduce the severity of COVID-19 infection through a similar mechanism. Because of these benefits, ECGC and green tea extract are also considered as potential antifibrotic agents [100]. The use of green tea polyphenols in the management of COVID-19 is shown in Figure 1.

## 5. Nanoparticle Drug Delivery System

Sucrose and ascorbic acid formulations have been demonstrated to improve catechin bioavailability by increasing bioaccessibility and intestinal tea absorption [102], as did vitamin C and xylitol formulations, which increased the transport rate of nongallated catechins by inhibiting the efflux transport mechanism [103]. Nanotechnological methods are among the most innovative and promising of these techniques. Nanotechnology has been proposed as an effective method for increasing EGCG bioavailability [104].

Bioactive compound nanoencapsulation is a simple and effective method for increasing the physical stability of bioactive compounds under gastrointestinal conditions, such as protecting compounds from contact with digestive components preventing premature degradation and excretion, which allows the compounds to be absorbed and can increase bioactivity. Compared with larger microparticles (>500 nm), biodegradable 100 nm nanoparticles (NPs) demonstrated a 15- to 250-fold enhancement in absorption [105]. NPs can also increase the total time that phytochemicals remain in circulation. Nanoencapsulation significantly improved EGCG stability and regulated distribution, which might potentially increase cellular EGCG uptake [106].

The use of NPs for drug delivery may increase bioavailability while decreasing chemopreventive agent toxicity [107]. Encapsulation is critical for increasing bioactive compound accumulation in food matrices. Encapsulation can also maintain separation between bioactive components and food additives [108]. Nanotechnology has been used to create a variety of delivery mechanisms for bioactive substances and nutraceuticals, in addition to providing protection and regulating distribution, which typically involves the encapsulation of compounds within NPs (radius <500 nm) [109].

NPs (10–1000 nm) containing bioactive compounds can be prepared using emulsification, coacervation, inclusion complexation, emulsification-solvent evaporation, nanoprecipitation, and supercritical fluid techniques [110]. Certain drugs/bioactive compounds are associated with insufficient oral bioavailability due to their extensive first-pass metabolic clearance. NP delivery systems can increase drug/bioactive compound levels by preventing presystemic hepatic metabolism [111]. By raising the gastric resistance time through enhanced mucosal adhesion [112] or increasing entry into cells or tissues (e.g., through Peyer's patches and M cell-mediated uptake) [113], NP-based delivery systems can increase oral drug/bioactive compound absorption.

Tea polyphenols packaged into NP formulations are less susceptible to harmful conditions in the GI tract, resulting in reduced enzymatic and nonenzymatic degradation and contributing to increased absorption into plasma. Encapsulating drug/bioactive molecules into NPs reduces the rate of plasma clearance, increases the apparent half-life of the drug/bioactive compound, and promotes the accumulation of the drug/bioactive molecule in target tissues [114]. In addition, encapsulating bioactive compounds in NPs can improve bioavailability by reducing transporter-mediated efflux.

Although nanodelivery networks have some advantages, they also have some drawbacks, such as the formation of NP aggregates, which can make the physical handling of NPs difficult in both liquid and dry forms. Due to the small particle size and the large surface area, the loading of drugs/bioactive molecules can be limited, and burst release can occur. A small number of studies have been conducted to determine whether EGCG can be protected from degradation and oxidation by NPs [115]. Studies examining oxidative degradation are especially beneficial because EGCG is highly susceptible to oxidation, particularly in alkaline environments. After entering the GI tract, NPs are subjected to a variety of pH conditions, excess ions, and digestive enzymes, which can affect the delivery of NPs and their cargo, including green tea polyphenols [116].

## 6. Bioavailability of Green Tea Polyphenols

Green tea polyphenols have been extensively studied as cancer prevention agents, and several in vitro studies have confirmed their high antioxidant activity. Additional in vivo studies remain necessary to investigate the pharmacokinetic relationship between absorption and the antioxidant function of green tea. When tea polyphenols were provided in capsule form as a green tea alternative, flavanol absorption increased, contributing to a small but significant increase in antioxidant activity in plasma compared to the delivery of polyphenols in green tea [117]. However, the observed increase in plasma flavanol concentration was not sufficient to increase the antioxidant capacity, suggesting that the observed increase in plasma antioxidant activity might be attributable to flavanol metabolites or degradation products [118].

The potency of EGCG was examined in in vitro studies at concentrations ranging from 1 to 100 mol/L; however, the peak plasma levels of tea catechins measured in human subjects or animals after the oral administration of green tea catechins are typically in the sub- to low-micromolar range [45], which is below the tested concentrations used in in vitro studies. Due to poor gastrointestinal stability and reduced permeability through the intestinal membrane, the medicinal benefits are also limited [119–121]. Poor systemic absorption due to low intestinal absorption, poor pharmacokinetics and bioavailability, poor biodistribution, first-pass metabolism, poor penetration, and low concentrations at targeted tissues limits the therapeutic potential of catechins. Despite the superior biological activity observed for catechins in vitro, the slow absorption rate in vivo may also be due to low stability, which can contribute to the formation of degradation products and prooxidant molecules.

Catechins are easily destroyed or metabolized under physiological conditions due to interactions with the hydroxyl groups on the phenol rings [122]. Even when catechins were delivered intravenously, they are often partially degraded before reaching target tissues [123]. The delivery of green tea polyphenol products in amounts equivalent to the EGCG contents found in 8–16 cups of regular green tea is thought to reduce the effects of poor bioavailability [124], and the systemic availability of EGCG was found to increase with increasing doses, likely due to the saturable presystemic elimination of orally administered green tea polyphenols [125]. The reported low bioavailability of green tea catechins is likely responsible for discrepancies between in vitro and in vivo studies, and stability, absorption rate, and efflux can all affect bioavailability. High pH conditions in the stomach and intestine and the effects of digestive enzymes contribute to catechin instability, degradation, and conjugation.

Nanostructure-based drug delivery systems may improve catechin bioavailability and allow for molecular modifications and the coadministration of other bioactive products. Tea catechins encapsulated in protein-based, carbohydrate-based, and lipid-based NPs demonstrated stability, sustained release, and permeation to the cell membrane, resulting in increased bioavailability. Molecular alterations, such as peracetylated EGCG (AcEGCG), can protect EGCG hydroxyl groups from oxidative degradation until deacylation occurs inside the cell [126], reducing biotransformation and EGCG efflux. The coadministration or formulation of catechins with other suitable medicinal products or bioactive compounds can yield synergistic effects, resulting in increased absorption and the inhibition of efflux transporters [127–130]. Tea polyphenols (TPPs) encapsulated in gelatin NPs (TPP-GNPs) were surface-modified with polyelectrolyte layers and characterized. The spherical NPs had a diameter of approximately 50 nm. The number of polyelectrolyte layers and the incubation time both had effects on encapsulation efficiency (EE). The highest EE was found for NPs featuring six polyelectrolyte layers (TPP-GNP-6L) and incubated for four hours. The evaluation of TPP release from TPP-GNP-6L in simulated biological fluids demonstrated that encapsulation provided TPP protection and allowed for controlled release. According to mathematical modelling, the anomalous form is the most common style of TPP release. TPP-GNP-6L had better pharmacokinetics than free TPP in a rabbit model. TPP-GNP-6L had a slightly higher area under the concentration-time curve and a longer mean residence time than free TPP, indicating that encapsulation increased TPP bioavailability. The researchers discovered that encapsulating TPP in GNPs resulted in a more consistent and prolonged TPP and improved the pharmacokinetics and bioavailability, which could help TPP last longer [131].

TPPs have low absorption and bioavailability due to their low solubility, poor durability, low permeability, and active efflux process in the gastrointestinal tract. The nanoencapsulation of TPPs using chitosan NPs can increase their stability and protect them from oxidation or degradation in the GI tract. Chitosan NPs may improve TPP absorption by disrupting near junctions or promoting direct uptake by epithelial cells via endocytosis. However, due to various factors, including pH, ions, digestive enzymes in the GI tract, and the mucus layer, which can influence the properties of NP delivery systems, the stability of chitosan NP must be more thoroughly evaluated. Future research should focus on the synthesis of TPP-encapsulated chitosan NPs that are suitable for oral administration, with improved GI stability and mucus penetrating activity, in addition to epithelial cell targeting properties [132].

## 7. Side Effects and Toxicity Associated with Green Tea Polyphenols

Green tea polyphenolic catechins have been demonstrated to have chemopreventive activity in a number of animal-tumor models, including the liver, urinary bladder, mammary gland, prostate, and hair [133–136]. The National Cancer Institute's Division of Cancer Prevention is currently testing a finely concentrated and standardized green tea extract (Polyphenon E®; PPE) as a candidate cancer chemopreventive agent, which is currently in Phase 2 clinical trials. Another laboratory has also completed 9-month chronic and 13-week subchronic follow-up PSA experiments in dogs. The doses used for the 9-month chronic trial (0, 200, 500, and 1000 mg/kg/day) were chosen based on an earlier 13-week subchronic oral toxicity analysis in dogs fed with a no observed adverse effect level (NOAEL) of >600 mg/kg body weight (highest dose tested) [137]. However, because of the poor oral bioavailability of green tea catechins [119, 138] and the improved oral bioavailability of free catechins after administration of PPE to human volunteers on an empty stomach [139], the chronic sample was delivered under fasting conditions. Unexplained elevations in morbidity and mortality resulted in the premature termination of the study at 6.5 months. The underlying causes of extreme toxicity were not determined during the chronic analysis due to high morbidity and mortality. A 13-week follow-up study was initiated to determine whether the results could be replicated in another laboratory and to identify and distinguish the toxicity of PPE after oral capsule dosing in dogs, in addition to investigating other potential variables, such as drug delivery under fed or fasting conditions.

In a 13-week oral toxicology trial in Beagle dogs treated with PPE two hours after feeding, NOAEL was greater than 600 mg/kg/day, the highest dose assessed. Following the report of significant improvements in PPE bioavailability in fasted human volunteers, the experiment was repeated using dosing under fasting conditions to maximize the biological effects of this agent [140], and long-term preclinical oral toxicology trials on fasted animals were conducted. PPE doses of 200, 500, and 1000 mg/kg/day were provided to fasting male and female Beagle puppies. Due to toxicity, including mortality in one specimen, the high dose was discontinued on day 9, and dosing was resumed 8 days later at 800 mg/kg/day. In contrast to the previous 13-week trial of fed Beagle puppies, which found no toxicity, all fasted dogs in the PPE-treated groups experienced significant morbidity and mortality. In 16 of 24 PPE-treated animals, unplanned mortality occurred, associated with clinical chemistry abnormalities and unusual coagulation and urinalysis parameters. The study was terminated prematurely after 6.5 months due to unnecessary morbidity and mortality. Multiple toxicities were identified in a large number of animals, including hematologic effects (decreased red blood cells, hemoglobin, hematocrit, white blood cells, neutrophils, monocytes, and platelet counts) and gross anatomical effects (lesions of the gastrointestinal tract, lymph nodes, liver, intestine, lungs, heart, and tonsils).

Even at significant doses, the oral consumption of green tea has not been associated with any negative effects and may be beneficial. An epidemiological study of over 8,500 individuals followed up for 9 years, for example, discovered that the age-standardized average annual cancer incidence rate was slightly lower among women who consumed at least 10 cups of green tea on a daily basis [137]. However, the effects of supplements have not been examined.

## 8. Conclusions and Future Directions

The COVID-19 pandemic has affected all facets of life, contributing to a thorough search for potential treatments against SARS-CoV-2, the causative virus underlying COVID-19. Although SARS-CoV-2 vaccines have been developed, effective COVID-19 antiviral drugs have not yet been developed. Because the pace of vaccination production and release has been limited, achieving complete herd immunity may take several years. In addition, new coronavirus diseases are likely to appear in the future. Many antiviral drugs should be developed for the treatment or alleviation of current and future coronavirus diseases.

Green tea polyphenols provide antiviral activity in various forms, which is important as researchers race to find COVID-19 antidotes under current pandemic conditions. EGCG, one of the most important catechins present in green tea, is increasingly viewed as a potential treatment agent for SARS-CoV-2 infection. Because most results from in vitro studies have been obtained using EGCG, animal testing or clinical testing is expected to validate the effects of EGCG against coronavirus disease. Because EGCG is the key component of green tea, green tea extracts can be used in in vivo studies. Because the safety of green tea has long been verified, a sufficient volume of green tea can be used in in vivo tests without toxicity concerns to determine whether green tea is beneficial for coronavirus diseases. Furthermore, an epidemiological analysis will be helpful for studying the impacts of green tea on coronavirus diseases. Data from epidemiological research should be analyzed to assess the impacts of green tea intake on the coronavirus, such as examining the association between the personal consumption of green tea and the likelihood of contracting coronavirus disease. We expect future research to focus on green tea polyphenols and to conduct detailed studies to validate the medicinal impacts of green tea on coronavirus diseases.

## Data Availability

The datasets supporting the conclusions of this study are included within the article (and its additional files).
